# Metabolomics and *in-vitro* bioactivities studies of fermented *Musa paradisiaca* pulp: A potential alpha-amylase inhibitor

**DOI:** 10.1016/j.heliyon.2024.e24659

**Published:** 2024-01-19

**Authors:** Adeleke Kazeem Atunnise, Olusola Bodede, Adewale Adewuyi, Vinesh Maharaj, Gerhard Prinsloo, Bamidele Adewale Salau

**Affiliations:** a Department of Biochemistry, Redeemer's University, Ede, Osun state, Nigeria; b Biodiscovery Center, Department of Chemistry, University of Pretoria, Pretoria, 0028, South Africa; c Department of Chemical Sciences, Redeemer's University, Ede, Osun state, Nigeria; d Department of Agriculture and Animal Health, University of South Africa, Florida, 1710, South Africa; e Antimicrobial Discovery Center, Department of Biology, Northeastern University, Boston, MA, USA

**Keywords:** Antioxidants, Diabetes, Fermentation, Metabolomics, Inflammation, Unripe-plantain pulp

## Abstract

The *in-vitro* synthesis of bio-compounds via fermentation is a promising route for bioactive molecules intended for disease control and management. Therefore, this study evaluated the effect of fermentation on the antioxidants, antihyperglycemic and anti-inflammatory properties and the resultant chemometric phytochemical profiles of unripe plantain fruits. The results revealed that *Escherichia coli* and *Propionibacterium* spp. are suspected as the key fermenters. The *E coli* showed negative results to the pathogenicity test*; Propionibacterium* appeared to be opportunistic. A significant increase in the total polyphenols and protein and decreased flavonoids was recorded in the phytochemical profile of the methanolic extract of the fermented unripe plantain pulp; however, the ascorbic acid content was not significantly altered. The ^1^H NMR fingerprint showed that there is a closely related chemical shift among the shorter fermentation time (days 2–6) and the unfermented, while the more extended fermentation periods (days 7–12) with enhanced bioactivities were closely related based on the chemometrics analyses. Furthermore, the UPLC-QTOF-MS analysis annotated the presence of bioactive compounds in the day-9 fermented sample: polyhydroxy glucose conjugates (3-Methoxy-4-hydroxyphenyl 6-*O*-(3,4,5-trihydroxybenzoyl)-beta-D-glucopyranoside), short chain peptide (leucyl-glycyl-glycine), amino acid derivatives (4-Aminophenylalanine, and N-Acetylhistidine), linear and cyclic fatty acid derivatives (palmitoyl putrescine, ricinoleic acid, phytosphingosine, gabalid, rubrenoic acid, 2-aminocyclopentanecarboxylic and cystodienioc acid). The synergistic effect of these newly formed compounds and the increase in the phenolic content of the day-9 fermented unripe plantain may account for its more potent antioxidant, anti-inflammatory and antihyperglycemic activity. Therefore, the products obtained from the day 9 fermentation of unripe plantain pulp may serve as potential nutraceutical agents against gastro-enteric sugar digestion and absorption and sugar-induced oxidative stress, inflammation and metabolic disease.

## 1 Introduction

Fermentation is one of the methods used for the biotransformation and degradation of biomass into value-added products through microorganisms' actions [1]. The method has shown capacity for synthesising bioactive compounds with health benefits in disease management [1,2]. Since the discovery and continued documentation of the health benefits of fermentation products, many natural products have been reported with profound benefits for humanity [1]. One of these benefits includes antidiabetic effects, which is of interest in this study.

Type II Diabetes Mellitus (T2DM) is a global challenge which requires immediate attention, especially in developing countries with little or no access to state-of-the-art medical care. T2DM is considered a heterogeneous metabolic disorder [3]. It is of serious concern because it claims many lives annually on a global scale, with studies indicating a continuous rise in the prevalence of the condition [4]. It is essential to develop a means for managing T2DM to reduce its prevalence and the danger it causes. Although several efforts have been reported using synthetic bioactive products from chemical-based molecules, these bioactive products from chemical-based molecules have side effects that limit their usage. Interestingly, studies have shown that bioactive compounds synthesised via fermentation of biomass are safer with no side effects when compared with bioactive compounds from chemical-based molecules [[5], [6], [7], [8]]. Apart from this, the bioactive products from the fermentation of biomass are environmentally friendly and affordable.

The recent increasing awareness regarding the two major categories of fermentation products – the probiotics and prebiotics, acting in the triad status of pharmaceutical, nutraceutical and nutritional supplements [1] is changing the dynamics of biomedical and chemical research as biomodified compounds are beginning to show substantial efficacies and sustaining health safety over their synthetic counterparts [9]. This is not unassociated with the synergy between fermented sample constituents - probiotics and probiotics (synbiotics). These synbiotic molecules have been reported to prevent and treat chronic diseases, including terminal ones like cancer and other neurodegenerative disorders, and have shown little or no side effects [9]. Hence, numerous plant products are recently fermented to generate biologically stable, active and non-toxic compounds to compete against their synthetic counterparts. Among these plants are pineapple, pawpaw, avocado, banana and plantain fruits [[9], [10], [11]]. Several biomasses have been identified as rich sources of bioactive compounds, including bioactive peptides, usually generated via the fragmentation of long-chain peptides during fermentation [12]. However, those studies focused on *Musa paradisiaca* (plantain) fruit flour as a source of bioactive compounds. Hence, fermented and unfermented unripe *Musa paradisiaca* (plantain) fruit flours are the most prescribed staple foods for aged, obese and diabetic patients in Nigeria and many parts of tropical Africa, India and Asia due to their nutraceutical profiles such as high insoluble fibre, minimal soluble sugar, bioactive free fatty acids and phytochemicals that act as signalling molecules in glycemic control, hormonal coordination, neural communications and other biochemical processes [2,[13], [14], [15]]. This nutraceutical profile of *Musa paradisiaca* is the reason for its use in this study. The nutritional composition of the unripe plantain fruit, like dietary fibre and other minerals, was claimed to be a significant player in many of these bioactivities and health benefits [15,16]. However, the multifactorial pathogenesis features of Type II Diabetes Mellitus (T2DM) and their associated risk factors [17,18] persist in the frontline of the global health burden list despite the several available remediation measures. Despite the abundant availability of *Musa paradisiaca* in many parts of the world, there is limited study on anti-T2DM study of its fermented bioactive compositions.

To our knowledge, the available therapeutical agents for managing T2DM present several complications except the ones of dietary therapy [19,20]. Thus, there is a need to explore the biomodification of plantain fruit to form new bioactive compounds with a more potent and safe profile against this multifactorial degenerative disease and its associated complications. This study aims to investigate the influence of extended liquid fermentation on unripe plantain pulp phytochemicals, antioxidant, anti-inflammatory and amylase inhibition potentials.

## 2 Materials and methods

### 2.1 Materials

All the apparatus used were laboratory grade, while chemicals and water were analytical grades. Unripe plantain fruits were freshly harvested at the Redeemer's University Botanical Garden, Ede, Osun state, Nigeria.

Gallic acid, quercetin, catechol and ascorbic acid were from Merck and Aldrich companies (Germany), while 1,1-diphenyl2-picrylhydrazyl (DPPH) was from Sigma Chemical Inc. (USA). Porcine pancreatic amylase and egg albumin were obtained from Ak-Scientific (USA). Other chemicals and solvents were analytic grades or otherwise synthetic grades.

#### 2.1.1 Sample preparation and methanolic extraction

The harvested unripe plantain fruit was peeled using a sterile knife, and the plantain pulp was homogenised in a sterile electric blender into a paste by adding sterile distilled water in a 1:1 (w/v) ratio. The paste was transferred into an isothermal batch-fermenting chamber. A fermented sample (750 g) was collected at intervals from the batch-fermenter every 24 h for 13 days. The collected sample was extracted with absolute methanol by agitating the fermented sample-methanol mixture (1:2, w/v) for 30 min at 150 rpm to allow maximum dissolution before filtering it with the Whatman No. 1 filter paper. The filtrate obtained was concentrated on the rotatory evaporator at 45 °C.

### 2.2 Phytochemical analyses

#### 2.2.1 Ascorbic acid content determination

To achieve this, 100 μg/mL of ascorbic acid (standard) ranging between 200 and 1000 μL and triplicate samples of 500 μL of the extracts were prepared. The volume was made up to 2000 μL with 4 % trichloroacetic acid. 500 μL of 2,4-dinitrophenyl hydrazine reagent (DNPH) was added to each tube, followed by two drops of 10 % thiourea solution. The mixture was allowed to stand for 3 h at 37 °C. Then, 2500 μL of chilled 85 % sulphuric acid was added and cooled on ice. The absorbance was read at 540 nm in a spectrophotometer (JENWAY 7305, Barloworld Scientific Ltd., UK). The ascorbic acid content was extrapolated from the ascorbic acid standard curve [24].

#### 2.2.2 Flavonoid content determination

Briefly, 500 μL of each extract (1 %) and four varying concentrations of quercetin standard (100 μg/mL) were mixed with 4 mL of vanillin reagent (1 % in 70 % sulphuric acid). Each tube was heated for 15 min in a boiling water bath, whereafter, the absorbance of each mixture was read on a spectrophotometer at 340 nm. The values of flavonoids were expressed as mg/g quercetin equivalent [25].

#### 2.2.3 The total phenols content determination

The extracts (500 μg/mL) and standard catechol solutions (0.2–1 mL) corresponding to 2.0–10.0 μg were pipetted out, and each tube was made up to 3.0 mL with distilled water, followed by adding 1 N Folin-Ciocalteau reagent (0.5 mL) and placed in a boiling water bath for precisely 1 min. The tubes were allowed to cool, and the spectrophotometer reading of the extracts was read at 650 nm. The phenolic content was deducted from the standard curve and was expressed as mg/g catechol equivalent [26]. .

#### 2.2.4 Total protein estimation

To determine this, 200–1000 μL egg albumin standards (100 μg/mL) and 100 μL of extracts (1 %) were pipetted into a series of test tubes and made up to 1.0 mL in all the tubes with distilled water. To this, 5.0 mL of alkaline copper solution ((50: 1 (v/v) sodium carbonate (2 % in 0.1 N NaOH) and copper sulphate (0.5 % in 1 % potassium sodium tartrate)) was added, thoroughly mixed and incubated for 10 min at 37 °C. Then 500 μL of 1 N Folin-Ciocalteau reagent was added, incubated for 30 min at 37 °C and read at 660 nm in a spectrophotometer [27].

### 2.3 ^1^H NMR (proton nuclear magnetic resonance spectroscopy) based metabolomics

##### 2.3.1.1 Acquisition of ^1^H NMR data

A 20 mg portion from each methanolic extract obtained from the 13 days of fermentation was dissolved in 500 μL of deuterium oxide (D_2_O) and transferred into a 5 mm NMR tube. The ^1^H NMR spectral data were obtained on a Bruker Avance III 400 MHz spectrometer equipped with a Double Resonance Broadband Probe (BBI) probe for optimum ^1^H signal resolution. The spectral acquisition was carried out in triplicate.

##### 2.3.1.2 Chemometric analysis

Before chemometric analysis, ^1^H NMR spectral data were processed using MestReNova (version 14.2.3, Mestrelab Research, Spain) software. Each ^1^H NMR spectrum was manually subjected to baseline and phase corrections, normalisation and alignment of peaks. The processed data were binned into 0.04 ppm bins representing spectrum range 0–10 ppm and then converted to Excel CSV file format for multivariate data analysis and pattern recognition. The transformed data were imported into SIMCA (soft independent modelling of class analogy) software, subjected to Pareto scaling and followed by the multivariate statistical analysis. An unsupervised statistical model, principal component analysis (PCA), was used to evaluate natural separation in the samples. In contrast, a supervised model, orthogonal projections to latent structure discriminant analysis (OPLS-DA), was employed to establish further the observed differences and patterns in the samples.

#### 2.3.2 Profiling of metabolites in day 9 extract using ultraperformance liquid chromatography-quadrupole time of flight-mass spectrometry (UPLC-QTOF-MS)

The day-9 extract exhibited the best bioactivity. Therefore, further studies were conducted on day-9 extracts for chemical identification and profiling. The separation of compounds in the day-9 extract and their detection was carried out using a Waters UPLC instrument hyphenated with a Waters Synapt G2 QTOF mass detector. The analysis was achieved in an Acquity UPLC BEH C18 1.7dμm (2.1 × 100 mm column), which operated at a 0.30 mL/min flow rate. The extract (5 mg) was dissolved in 1 mL 100 % LC grade water and filtered through a 0.22 μm syringe filter. The mobile phase used was: A, 0.1 % HCO_2_H in LC grade water and B, MeOH +0.1 % HCO_2_H. The MS source, ESI, was operated in a positive ion mode while the capillary and endplate voltage were set at 2600V and 2000 V, respectively. Nitrogen was employed as a nebulising gas set at 10 L/h while *m/z* range was set from 50 to 1200 amu. Gradient elution started with 97 % A and 3 % B, which remained linear until 14 min. From 14 to 16 min, elution was kept constant with 0 % A and 100 % B. A linear gradient of 97 % A and 3 % B was afterwards used to reach completion until 20 min. The chromatogram and the MS data were processed using MassLynx v 4.1 (Waters Corporation, Milford, MA, USA) software, which lists possible elemental formulae. The accuracy for confirmation of the compounds was established based on their mass error of less than 5 ppm and MS/MS fragment matching from relevant libraries and databases.

### 2.4 Microbial analyses

#### 2.4.1 bacteria cell count

The viable cell count was done on nutrient agar (NA) and prepared according to the manufacturer's instructions for isolating bacteria. At every 24 h for 13 days, samples were aseptically withdrawn and serially diluted for the isolation of bacteria. Colonies forming units formed on the media were counted and identified [28,29].

#### 2.4.2 In-vitro antioxidants analyses

##### 2.4.2.1 *1,1-diphenyl2-picrylhydrazyl*" (DPPH) spectrophotometric assay

The gallic acid (standard), ascorbic acid and extracts (20 μL) were added to 500 μL of 0.3 mM methanolic solution of DPPH and 480 μL of methanol. The mixture was allowed to react in the dark for 30 min at room temperature [30]. The extent of discolouration of the purple-coloured solutions was read at 518 nm in a spectrophotometer, and the radical scavenging activity was calculated as below:

$$Scavenging\;activity\;\%=100-\frac{Abs\;\left(sample\right)-Abs\;\left(blank\right)}{Abs\;\left(blank\right)}\;X\;100$$

#### 2.4.3 Ferric-reducing antioxidant power (FRAP)

Briefly, the FRAP of the extracts, ascorbic acid and standard (gallic acid) was determined by adding the 2.5 mL aliquot of the extracts to 2.5 mL of 200 mM (pH 6.6) sodium phosphate buffer and 1 % potassium ferricyanide (2.5 mL). The mixture was incubated for 20 min at 50 °C, adding 2.5 mL trichloroacetic acid (10 %) and centrifuging for 10 min at 2000×*g*. Exactly 5 mL of the supernatant was mixed with an equal volume of distilled water and 1 mL of ferric chloride (0.1 %) [31]. The absorbance was measured at 700 nm in a spectrophotometer (JENWAY 7305), and the radical scavenging activity was calculated as below:

$$Inhibition\;\%=\frac{Abs\;\left(sample\right)-Abs\;\left(control\right)}{Abs\;\left(control\right)}\;X\;100$$

#### 2.4.4 Hydrogen peroxide scavenging effects

A solution of H_2_O_2_ (40 mM in phosphate buffer) was added to 1 mg/μL of the extracts (600 μL) and made up to the total volume of 3 mL. The same was repeated for the standards (100 μL/mL gallic acid and ascorbic acid with volumes of 100–20 μL). A blank solution containing phosphate buffer, without H_2_O_2_, was prepared. The absorbance of the reaction mixture, blank, and standards was recorded at 230 nm in a spectrophotometer and calculated as and equated to gallic acid on the gallic acid standard curve [32].

$$Hydrogen\;peroxide\;scavenging\;\%=\frac{Abs\;\left(control\right)-Abs\;\left(sample\right)}{Abs\;\left(control\right)}\;X\;100$$

#### 2.4.5 Lipid peroxidation inhibition

The method was slightly modified, mixing 20 % liver homogenate (healthy rat liver homogenate obtained from Redeemer's University animal house) with ice-cold KCl (0.15 M). The extracts (1 mL) and the liver homogenate (1 mL) were mixed with 1 mL of a solution containing FeSO_4_ (25 mM), ascorbate (100 mM) and KH_2_PO_4_ (10 mM). The volume was made up to 3 mL with distilled water and incubated for 30 min at 37 °C. A 0.5 mL phosphate buffer (0.12 M, pH 7.2) was added to 0.5 mL of the homogenate solution, followed by the addition of 1.0 mL of (10 %) TCA and 1.0 mL of (0.1 M) and mixed thoroughly. The mixture was heated in a water bath at 80 °C for 20 min. The tubes were centrifuged at 1000×*g* for 10 min, and the spectrophotometer reading was taken at 535 nm against a blank containing all the reagents except the homogenate. The MDA equivalents of the samples were calculated using the extinction coefficient 1.56 × 105 M^−1^cm^−1^ [33].

#### 2.4.6 Egg albumin denaturation assay

A 200 μL of 1 % egg albumin solution, 2 mL of sample extract or standard (NSAID), and 2.8 mL of (pH 7.4, 0.2 M) phosphate-buffered saline were mixed and incubated at 37 °C for 30 min, followed by heating in a water bath at 70 °C for 15 min. The volume of the extract was replaced by distilled water in the control test tube. The reaction mixtures were read at 280 nm in the spectrophotometer, and percentage inhibition was calculated as stated below [34]:

$$Protein\;denaturation\;inhibition\;\%=\frac{Abs\;\left(sample\right)-Abs\;\left(control\right)}{Abs\;\left(control\right)}\;X\;100$$

#### 2.4.7 Measurement of nitric oxide scavenging activity

The reaction was initiated by adding 2.0 mL of sodium nitroprusside (100 mM), 500 μL of 0.2 M phosphate-buffered saline (pH 7.4) and 500 μL of each of the extracts (50 mg/mL), gallic acid (100 mg/mL) and ascorbic acid (100 mg/mL) in their respective test tubes and incubated for 30 min at 25 °C. A 500 μL Griess reagent (1 % sulphanilamide, 0.1 % naphthyl ethylene diamine dihydrochloride and 2 % H_3_PO_4_) was added and incubated at room temperature for another 30 min. Control tubes were prepared in the same manner without the extracts, and absorbance was read at 546 nm against the reagent blank in a spectrophotometer [35].

#### 2.4.8 In-vitro alpha-amylase inhibition assay

In brief, 100 μL of the extract was added to 200 μL α-amylase enzyme (Ak Scientific Inc. USA. Alpha-Amylase 7705EQ) solution (2 mM of phosphate buffer, pH-6.9) and 100 μL phosphate buffer and allowed to react for 20 min. After the incubation, 100 μL of 1 % starch solution was added and incubated for 5 min. The same was repeated for the controls, where 200 μL of the enzyme was replaced with the phosphate buffer. After incubation, 500 μL of dinitrosalicylic acid reagent was added to both extracts and control contents and boiled for 5 min in a water bath, after which they were allowed to cool down. The absorbance was read at 540 nm using a spectrophotometer [36].

$$Inhibition\;\%=\frac{Abs\;\left(sample\right)-Abs\;\left(control\right)}{Abs\;\left(control\right)}\;X\;100$$

#### 2.4.9 Statistical methods

Data generated were analysed with GraphPad Prism 8.0 version for Windows. The triplicate of values was subjected to analysis of variance (ANOVA), Turkey for the group comparison at p < 0.05 and Pearson correlation *heatmap* was used to evaluate correlation coefficients.

## 3 Results

### 3.1 Screening of bacterial fermenters

**Fig. 1:**
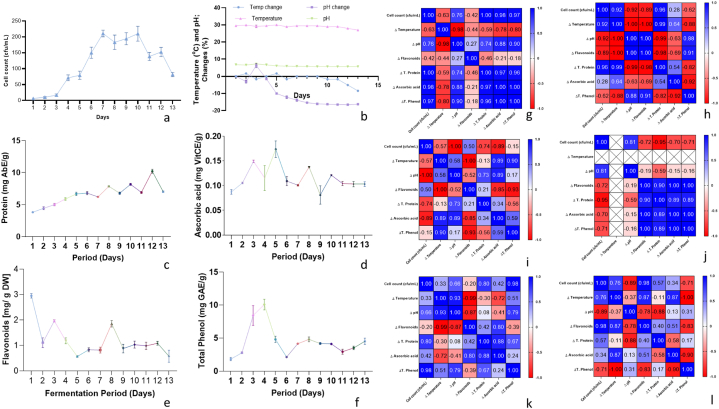
Effect of Fermentation on the Temperature, pH, phytochemicals and Pearson's correlation coefficients (*Heatmap*) of percentage change in phytochemical per fermentation intervals of Unripe Plantain Fruits Bacteria and Physicochemical changes: (a) Bacterial growth rate; (b) Changes in temperature and pH of fermented plantain; (c) Total protein content of fermented plantain sample; (d) Ascorbic acid content of fermented plantain sample; (e) Flavonoids content of fermented plantain sample; (f) Total phenolics content of fermented plantain sample. Correlations of fermenters growth with physiochemical changes at a 3-day Interval: (g) Days 1–3, (h) Days 3–5, (i) Days 5–7, (j) Days 7–9, (k) Days 9–11, (l) Days 11–13.

Fig. 1(a–f) shows the effect of fermentation time on the fermenter's proliferation (10^6^ X cfu/mL) and their resultant actions on the physiochemical parameters of the unripe plantain pulp. Fig. 1 (g – l) was the Pearson's correlation coefficients between the proliferation rate of the fermenters and the physiochemical of the methanolic extract of the fermented plantain pulp at three days intervals.

Fig. 1a showed that the proliferation rate of the plantain pulp fermenters was not significant (p > 0.05) between days 1 and 3; a drastic increase in the rate was noted from days 4–6, and a plateau was recorded from days 6–10; while a decline in the proliferation rate of the fermenters was observed at day 11. The decline in the bacteria growth persisted till the end of this study.

### 3.2 Effect of fermentation on the phytochemical profile of unripe plantain fruit

Fig. 1b presents the temperature and pH changes of unripe plantain pulp; these factors correlate with bacteria viability in a fermenting sample. The temperature and pH values of the fermented samples from days 1–13 ranged from 30 °C to 27 °C and 82 to 5.71, respectively. Also, the decrease in temperature was not pronounced until day 11, and the pH change started declining from day 4. Fig. 1c showed a steady increase in the protein content from day 1–6. In Fig. 1d, the ascorbic content was at its peak at day 5 and, the least was in the unfermented (day 1). The highest flavonoids and the least were observed on days 1 and 13, respectively, in Fig. 1e. In Fig. 1f, phenolic content was at its peak on day 4, but the least was in the unfermented.

Pearson correlation *heatmap* are present in Fig. 1g to l. In Fig. 1g, there is a positive association between the bacteria proliferation rate, protein (p-value: 1.00), ascorbic acid (p-value: 0.98) and phenolics (p-value: 0.97) contents within the first three days of the fermentation period. In Fig. 1h–a positive association (p-value: 0.96) was observed between the bacteria proliferation and the protein content of the fermented pulp methanolic extract up to day 5. From day 5–7, the pH changes negatively correlated (p-value: 1.00) with the bacteria proliferation rate in Fig. 1i. In Fig. 1j–a significant negative correlation (p-value: 0.96) was observed between the flavonoid content and the bacteria proliferation rate at days 7–9. On days 9–11, a positive correlation (p-value: 0.98) was recorded between the flavonoid content and bacteria proliferation rate, Fig. 1j. In Fig. 1k (days 9–11) and Fig. 1l (days 11–13), the proliferation patterns of the bacteria at each interval are positively correlated with phenols (p-value: 0.98) and flavonoids (p-value: 0.98) contents, respectively.

### 3.3 ^1^H NMR-based metabolomics

#### 3.3.1 Chemometric analysis

**Fig. 2:**
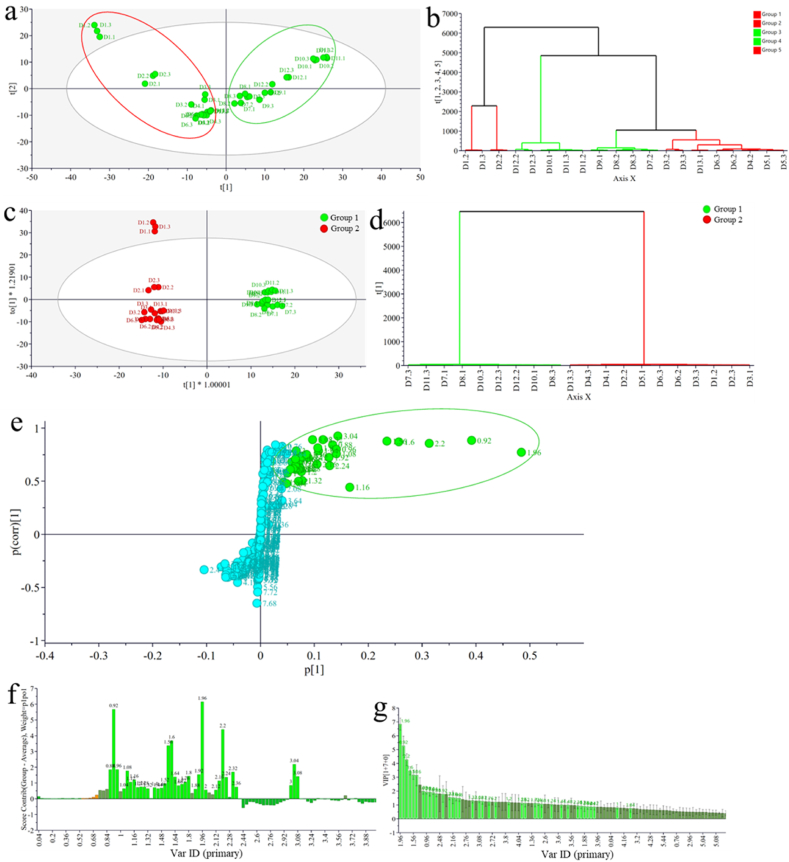
PCA (a: score plot, b: HCA dendrogram) and OPLS-DA (c: Score plot, d: HCA dendrogram, e: S-plot, f: contribution plot, g: VIP scores) of fermented plantain extracts. The light green spheres and bars represent chemical shifts (ppm) of the region of the S-plot, contributing to the separation of group 1 (light green scores) from group 2 (red scores).

An exploratory approach was adopted to understand the pattern of metabolite distribution across the thirteen-day fermentation period. The PCA score plot presented in Fig. 2a showed a natural unsupervised pattern in the samples. The PCA model had a goodness of fit (R^2^X_(cum)_) value of 0.962 and a predictability (Q^2^_(cum)_) value of 0.928. Two broad groups were observed, with samples from day 1 to day 6 and day 13 separated into the left quadrants (red sphere), while samples from day 7 to day 13 separated into the right quadrants (green sphere). However, day 1 was observed as outliers forming a cluster outside the 85 % confidence interval, whereas day 2 formed separate clusters on the left and days 10, 11 and 12 were more pronounced on the far right. The first two PCs described 78.3 % of the total variance in the samples, with PC1 and PC2 describing 57.1 % and 21.2 %, respectively. The separation of the samples in the PCA was confirmed as five groups were observed on the hierarchical cluster analysis (HCA) dendrogram (Fig. 2b). The groups are as follows: days 1 and 2 as group 1 and 2, respectively; days 10–12 (group 3); days 7–9 (group 4); and days 3–6 and 13 (group 5).

To further understand the separations in the two broad groups observed using PCA, an OPLS-DA model was created, giving rise to the various explorative plots presented in Fig. 2c – g. The goodness of fit (R^2^X_(cum)_; R^2^Y_(cum)_) for the OPLS-DA model were 0.979 and 0.989, whereas its predictability (Q^2^_(cum)_) was 0.971. The score plot (Fig. 2c) showed a clear separation between groups 1 and 2; day-1 samples persisted as outliers similar to the PCA. The HCA dendrogram further confirmed the separation of the samples into two groups (Fig. 2d). The loading S-plot (Fig. 2e) and contribution plot (Fig. 2f) were generated to reveal the significant discriminant NMR bins of the two groups. Although these two plots are complementary, the S-plot provides additional detail for the discriminant NMR chemical shifts as the group 1 associated scores on the plot separate farther away from the center of the S towards the right. In contrast, those of group 2 are separated to the left. Group 1 was explored further based on extended fermentation criteria. With respect to the OPLS-DA results, the variable importance in the projection (VIP) was generated (Fig. 2g). The VIP scores were arranged in decreasing order of significance from left to right. Furthermore, the chemical shifts contributing to the separation of Group 1 from Group 2 were distinguished using the light green colour code, as indicated in the contribution plot. For VIP score ≥1.5, the NMR region consists of 1.96, 0.92, 2.2, 1.6, 1.56, 1.16, 1.08, 0.96, 2.24, 3.04, 0.88, 1.92, 2.32, 2.16 and 1.64 ppm bin values. Those that fall between 1 and 1.5 include 1.8, 1.32, 3.08, 1.12, 1.76, 2, 1.2, 1.28, 1.52, 1.72, 1.4, 1.68, 1.24 and 1.44 ppm, while those with VIP scores less than 1 were considered insignificant.

#### 3.3.2 Compounds annotation from ^1^H-NMR fingerprint supported by chemometric findings

**Fig. 3:**
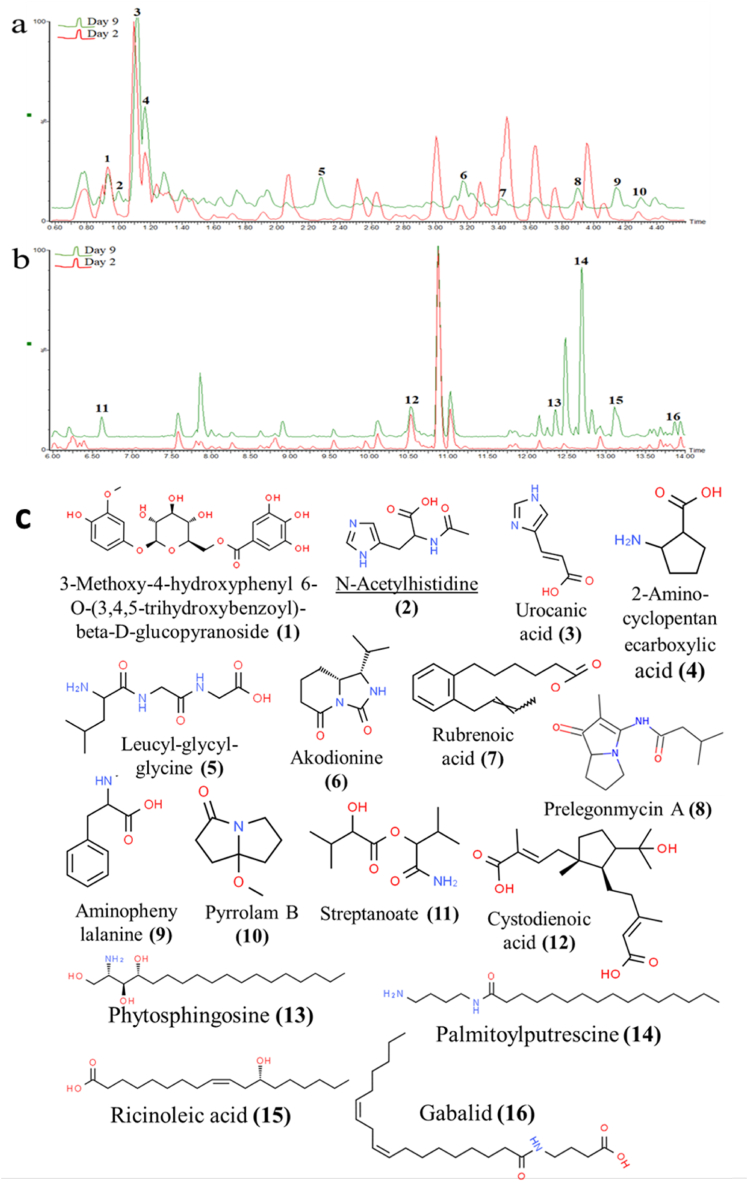
UPLC-QTOF-MS chromatogram (expanded) of day 2 and 9 samples of the fermented unripe plantain fruits and annotated metabolites: (a) expanded between 0.6 & 4.5 min, (b) expanded between 6.0 & 14.0 min, (c) structures of annotated compounds.

Chenomx software was employed alongside the Human Metabolome Database (HMDB) database and relevant literature to detect compounds with characteristic chemical shifts that distinguished group 1 from group 2. For comparison, the ^1^H NMR spectra of day 9 and day 2 were selected from groups 1 and 2, respectively. The highly deshielded region of the ^1^H NMR spectra showed a higher number of peaks on day nine than on day 2. In contrast, day 2 had more peaks with relatively higher intensities in the sugar region. However, the most distinguishing region of the ^1^H NMR spectra was between 0.5 and 3.3 ppm, which was confirmed by the contribution plot and VIP scores generated from group 1 of the OPLS-DA cluster analysis (Fig. 2g). The full and expanded ^1^H NMR spectra are provided in the supplementary material (Fig. 3). The possible compounds responsible for the better bioactivity of day 9 (group 1) were then identified based on their respective characteristic chemical shifts. The identified compounds include the two phenolic acids, gallic acid alongside its methyl ester and ferulic acid; primary amino acids, alanine, leucine and l-acetylleucine, a monosaccharide sugar, mannose and four lipids; ricinoleic acid, coriolic acid, N-acetyl dihydrosphingosine and palmitoyl putrescine (Table 1).

**Table 1:** Diagnostic ^1^H NMR chemical shifts of compounds identified from NMR fingerprints of fermented unripe plantain fruits.

**S/N**	**Compound**	**Chemical shift (ppm)**		**Reference**
		Current study	Reference value	
1	Gallic acid	7.25	7.22	[37]
2	Methyl gallate	3.95, 7.26	4.01, 7.26	[37]
3	Alanine	1.49, 3.76	1.47, 3.77	HMDB
4	Leucine	1.04, 1.61, 3.70,	0.95, 1.70, 3.72	HMDB
5	l-Acetylleucine	0.89, 0.91, 1.57, 1.59, 1.99, 3.95 8.05 (NH)	0.88, 0.92, 1.58, 1.60, 1.70, 2.02, 3.95	HMDB
6	Mannose	1.15, 3.35, 3.57, 3.70, 3.85, 5.19	1.20, 3.37, 3.50, 3.68, 3.84, 4.77, 5.01	[38]
7	Ricinoleic acid	0.85, 1.18, 1.53, 2.00, 2.17, 2.20, 3.45, 5.47	0.80, 1.17–1.54, 2.10, 2.19, 2.22, 3.53, 5.35, 5.46	[39]
8	Coriolic acid	0.89, 1.20, 1.35, 1.51, 1.59, 2.22, 2.30, 3.90, 5.33, 5.36, 5.98, 6.43	0.91, 1.25–1.40, 1.54, 1.60, 2.20, 2.27, 4.08, 5.41, 5.62, 5.98, 6.50	[40]
9	N-acetyl dihydrosphingosine	0.89, 1.21, 1.56, 2.20, 3.73, 3.91	0.88, 1.25, 1.61, 2.23, 2.79, 3.80, 4.01	[39]
10	Palmitoylputrescine	0.91, 1.30, 1.55, 2.17, 2.99, 3.23, 8.46 (NH)	0.90, 1.20–1.40, 1.56–1.64, 1.65, 2.18, 2.94, 3.20	[41]

HMDB: Human Metabolome Database [42].

#### 3.3.3 Ultraperformance liquid chromatography-quadrupole time of flight-mass spectrometry (UPLC-QTOF-MS) analysis

Ultraperformance liquid chromatography-quadrupole time of flight-mass spectrometry, being a more sensitive technique (compared to ^1^H NMR), UPLC-QTOF-MS was further employed for metabolite profiling. Sixteen compounds were identified, and detailed reports are provided in Table 2. Fig. 3a and b shows comparative chromatograms of days 2 and 9 while the structures of the compounds are presented in Fig. 3c. It can be inferred that the UPLC-QTOF-MS report validated the findings of the NMR based on structural similarities in their identified compounds. It was observed that there were more early elution peaks (Rt 0.8–4.5 min) on day 2 compared to day 9. The chromatograms also revealed that there were more late elution peaks (Rt 10.0–14.0 min). Moreover, nine of the sixteen compounds were found unique to day 9. These include N-acetylhistidine, leucyl-glycyl-glycine, akodionine, 4-aminophenylalanine, pyrrolam B, streptanoate, phytosphingosine, palmitoylputrescine and ricinoleic acid.

**Table 2:** Report of compounds identified based on UPLC-QTOF-MS of fermented unripe plantain fruits.

S/N	**Rt (min)**	**Observed mass (m/z)**	**Calculated mass (m/z)**	Mass error (ppm)	Adduct	MS/MS fragment Ions (*m*/*z*)	Molecular Formula	Compound	CAS-RN
1	0.93	455.1172	455.1190	−4.0	[M+H]^+^	439.5380	C_20_H_22_O_12_	3-Methoxy-4-hydroxyphenyl 6-*O*-(3,4,5-trihydroxybenzoyl)-beta-D-glucopyranoside	109,194-55-0
						186.1019			
						176.0904			
						127.0768			
2	1.00*	198.0877	198.0879	−1.0	[M+H]^+^	178.1088	C_8_H_11_N_3_O_3_	N-Acetylhistidine	10,101-30-1
						156.0888			
						136.0459			
3	1.12	139.0508	139.0508	0.0	[M+H]^+^	121.0472	C_6_H_6_N_2_O_2_	Urocanic acid	104-98-3
						95.0669			
						93.0501			
4	1.16	130.0870	130.0868	1.5	[M+H]^+^	114.0517	C_6_H_11_NO_2_	2-Aminocyclopentanecarboxylic acid	3814-46-8
						84.0845			
5	2.28*	246.1446	246.1454	−3.3	[M+H]^+^	201.1109	C_10_H_19_N_3_O_4_	Leucyl-glycyl-glycine	1187-50-4
						171.0881			
						87.0474			
6	3.17*	197.1287	197.1290	−1.5	[M+H]^+^	100.0805	C_10_H_16_N_2_O_2_	Akodionine	1,698,877-13-2
7	3.41	269.1497	269.1517	−7.4	[M+Na]^+^	232.1976	C_16_H_22_O_2_	Rubrenoic acid	1,006,037-84-8
						203.6295			
						137.1152			
8	3.90	237.1596	237.1603	−3.0	[M+H]^+^	151.1295	C_13_H_20_N_2_O_2_	Prelegonmycin A	1,802,335-50-7
						87.0493			
9	4.14*	181.0974	181.0977	−1.7	[M+H]^+^	135.0951	C_9_H_12_N_2_O_2_	4-Aminophenylalanine	943-80-6
						118.0706			
10	4.29*	156.1025	156.1025	0.0	[M+H]^+^	126.0567	C_8_H_13_NO_2_	Pyrrolam B	151,680-43-2
						113.0869			
11	6.62*	240.1205	240.1212	−2.9	[M+Na]^+^	172.1069	C_10_H_19_NO_4_	Streptanoate	1,884,384-34-2
12	10.53	353.2296	353.2328	−9.1	[M+H]^+^	295.2355	C_20_H_32_O_5_	Cystodienoic acid	1,802,726-75-5
						277.2204			
13	12.36*	318.3008	318.008	0.0	[M+H]^+^	300.3004	C_18_H_39_NO_3_	Phytosphingosine	554-62-1
						282.2905			
						270.2881			
14	12.69*	327.3374	327.3375	−0.3	[M+H]^+^	310.3249	C_20_H_42_N_2_O	Palmitoylputrescine	126,617-68-3
						256.2773			
						239.2493			
15	13.11*	321.2402	321.2406	−1.2	[M+Na]^+^	306.3426	C_18_H_34_O_3_	Ricinoleic acid	141-22-0
						185.1788			
16	13.87	366.3006	366.3008	−0.5	[M+H]^+^	321.4270	C_22_H_39_NO_3_	Gabalid	84,393-31-7
						263.2411			

HMDB: Human Metabolome Database, *Compounds unique to day 9.

### 3.4 Morphological and Biochemical analysis of suspected bacteria isolated from the fermented unripe plantain pulp

Tables 3a, 3b and 3c present the morphological and clinical relevance, enzyme activities, and carbon source utilisation analyses of the two bacteria suspected to be responsible for the fermentation of the unripe plantain pulp for 13 days. The two suspected bacteria identified from the data in Table 3 are *E. coli* and *Propionibacterium species*. The data in Table 2a shows that both bacteria are creamy, rod-like in shape, non-pathogenic and weak lactose fermenters. Gram-staining results showed that the *E. coli* is gram-negative while the *Propionibacterium spp*. is gram-positive. In Table 3b, both bacteria showed similar responses to about 60 % of the 20 enzyme activity analyses in this study. The oxidative fermentation analysis showed that *Propionibacterium spp*. was a non-oxidative fermenter, while *E. coli* was positive to this test. Table 3c shows that both fermenters had the same responses to 50 % of the 21 carbon sources. However, *E. coli* tested positive for utilising 14 of the 21 carbon sources analysed; *Propionibacterium spp*. was positive for 10 of the 21 carbon sources.

**Table 3:** Morphological and biochemical characteristics of bacteria isolated from fermented sample.

Table 3a. Morphology and pathogenic screening of Fermenters			
Characteristics	*Escherichia coli*	*Propionibacterium* spp.	Remarks
Colour	Cream	Cream	
Gram Staining	-ve	+ve	
Shape	Rod	Rod	
Motility	+ve	-ve	
Anaerobic Tryptone Soya Agar	-ve	+ve	anaerobic organisms.
Cetrimide agar	-ve	-ve	No *Pseudomonas aeruginosa*
Nutrient agar for proteus spp.	No swirming	No swirming	No *Proteus* sp
Thiosulfate–citrate–bile salts–sucrose agar, or TCBS agar	No growth	No growth	No *V. cholerae* and *V. parahaemolyticus*
Mannitol Salt Agar	No growth	No growth	No pathogenic *Staphylococci*
MacConkey agar	+ve	Weak	Lactose fermenters
SS Agar (Salmonella Shigella Agar)	Weak	Weak	Lactose fermenters

Table 3b. Enzymes activity of Fermenters		
Enzymes activities characteristics	*E. coli*	*Propionibacterium* spp.
Acid Phosphatase	Variable	+ve
Alkaline Phosphatase	+ve	-ve
Arginine Dehydrolase	-ve	-ve
Catalase	+ve	+ve
Lecithinase	-ve	-ve
Lipase	-ve	-ve
Lysine decarboxylase	+ve	-ve
Ornithine decarboxylase	-ve	-ve
Oxidase	-ve	+ve
Phenylalanine deaminase	-ve	+ve
Tryptophan Deaminase	+ve	+ve
Urease	-ve	-ve
H_2_S	-ve	+ve
Indole	+ve	+ve
MR (Methyl Red)	+ve	+ve
Nitrate Reduction	+ve	+ve
OF (Oxidative/Fermentative)	+ve	-ve
ONPG (*o*-nitrophenyl-β-D-galactopyranoside)	+ve	+ve
VP (Voges–Proskauer)	-ve	-ve

Table 3c. Carbon sources utilisation of Fermenters		
Carbon source characteristics	*E. coli*	*Propionibacterium spp.*
Acetate Utilisation	+ve	-ve
Arabinose	+ve	+ve
Cellobiose	-ve	-ve
Citrate	-ve	+ve
Fructose	+ve	+ve
Galactose	-ve	+ve
Glucose	+ve	+ve
Glycerol	+ve	+ve
Glycogen	-ve	-ve
Inositol	-ve	-ve
Inulin	+ve	-ve
Lactose	+ve	Weak
Maltose	+ve	-ve
Mannitol	+ve	-ve
Mannose	+ve	+ve
Raffinose	-ve	-ve
Ribose	-ve	+ve
Sorbitol	+ve	+ve
Starch	+ve	-ve
Sucrose	+ve	-ve
Xylose	+ve	-ve

Keynotes: Positive; +ve; Negative: ve.

### **3.5** *Antioxidants, anti-inflammatory and alpha-amylase inhibition profile of Methanolic extract of unripe plantain pulp*

The data in Table 4 presents the effect of fermentation on antioxidant, anti-inflammatory and alpha-amylase inhibition on unripe plantain pulp. The DPPH IC_50_ value of fermented samples was significantly different (p < 0.05) from the unfermented (day 1) except for days 6, 10 and 11. The ferric-reducing antioxidant power of the fermented sample was not significantly different (p > 0.05) from unfermented samples except for days 2, 8, and 12, which had significantly (p < 0.05) high IC_50_ values. The hydrogen peroxide scavenging potential of the fermented and unfermented samples was not significantly different (p < 0.05). The lipid peroxidation inhibition (IC_50_) of days 8 and 9 was significantly lower (p < 0.05) than unfermented and the other fermented days. The protein denaturation inhibition (IC_50_) value of days 2 and 3 was significantly lower than day 1; however, days 4–12 were not significantly different (p > 0.05) from day 1. The IC_50_ of day 13 was significantly higher (p < 0.05) than of day 1. The nitric oxide scavenging IC_50_ value of days 9 and 11 was significantly (p < 0.05) lower than day 1; however, day one and other fermented days were not significantly different (p > 0.05). Alpha-amylase inhibition EC_50_ value of days 9 and 10 was significantly higher than day 1 (unfermented), while day 2 had a significantly higher EC50 value (p < 0.05). The alpha-amylase inhibition EC_50_ value of days 3–8 and days 11–13 was not significantly different from the unfermented (day 1).

**Table 4:** Effect of fermentation on the antioxidant, anti-inflammatory and α-amylase inhibition profiles of unripe plantain fruit samples.

Fermentation Period (Days)	DPPH Inhibition	Ferric Reducing Antioxidant Power	H_2_O_2_ Scavenging	Lipids Peroxidation Inhibition	Protein denaturation inhibition	Nitric oxide scavenging	α-amylase activity inhibition
	IC_50_ (μg/mL GAEq)	IC_50_ (μg/mL GAEq)	IC_50_ (μg/mL GAEq)	IC_50_ (μg/mL GAEq)	IC_50_ (μg/mL GAEq)	IC_50_ (μg/mL GAEq)	EC_50_ (μg/mL GAEq)
1	33.18 ± 0.80	20.83 ± 1.29	17.45 ± 1.54^ǂ^	41.03 ± 2.54	38.76 ± 5.23	24.85 ± 0.10	30.23 ± 1.41
2	27.26 ± 0.89*^ǂ^	28.67 ± 0.97*	20.47 ± 1.44^ǂ^	40.86 ± 2.60	29.05 ± 9.50*^ǂ^	25.13 ± 1.09	42.49 ± 1.40*
3	25.43 ± 0.92*^ǂ^	26.46 ± 1.01	19.64 ± 1.46^ǂ^	44.14 ± 2.19	28.85 ± 9.33*^ǂ^	23.63 ± 0.14	36.75 ± 1.32
4	23.25 ± 1.17*^ǂ^	22.37 ± 1.22	21.04 ± 1.40^ǂ^	43.55 ± 2.30	35.25 ± 6.66^ǂ^	22.61 ± 1.20	33.17 ± 1.34
5	23.31 ± 1.12*^ǂ^	22.40 ± 1.22	16.15 ± 1.64^ǂ^	43.11 ± 2.30	37.44 ± 6.10	23.38 ± 1.17	33.69 ± 1.43
6	26.77 ± 1.03^ǂ^	19.69 ± 1.36	19.35 ± 1.47^ǂ^	41.86 ± 2.45	37.29 ± 6.28	21.50 ± 1.29	33.56 ± 1.44
7	25.90 ± 0.95*^ǂ^	22.66 ± 1.23	17.04 ± 1.58^ǂ^	35.76 ± 1.17	45.58 ± 4.24	23.62 ± 1.14	29.72 ± 1.42
8	25.51 ± 1.08*^ǂ^	28.50 ± 0.96*	20.62 ± 1.41^ǂ^	36.10 ± 0.19*^ǂ^	42.63 ± 4.53	24.67 ± 1.14	32.14 ± 1.44
9	23.84 ± 1.15*^ǂ^	23.18 ± 1.19	19.02 ± 1.52^ǂ^	36.50 ± 0.12*^ǂ^	41.21 ± 4.51	22.18 ± 0.24*^ǂ^	24.67 ± 0.39*^ǂ^
10	28.93 ± 0.92^ǂ^	23.05 ± 1.18	23.75 ± 1.27	40.28 ± 2.73	39.39 ± 5.24	26.05 ± 1.07	24.61 ± 0.40*^ǂ^
11	25.98 ± 1.06^ǂ^	27.54 ± 0.98	22.09 ± 1.30	39.25 ± 0.02	43.99 ± 4.44	21.93 ± 0.24*^ǂ^	25.72 ± 1.06^ǂ^
12	23.85 ± 1.08*^ǂ^	33.41 ± 0.81*^ǂ^	22.30 ± 1.35	41.54 ± 2.55	41.07 ± 4.53	21.97 ± 0.22	29.72 ± 1.35
13	24.03 ± 1.00*^ǂ^	27.89 ± 0.96	21.24 ± 1.40^ǂ^	41.03 ± 2.66	47.19 ± 3.36*	21.41 ± 1.26	31.87 ± 1.37
Ascorbic acid	38.05 ± 0.79*	24.21 ± 1.11*	29.57 ± 0.98*	39.64 ± 1.78	42.85 ± 4.38	24.68 ± 0.12	
Acarbose (amylase inhibitor)	–	–	–	–	–	–	35.78 ± 1.37*

Values are expressed as Mean ± SEM for four replicate samples.

Values with superscript (*) are significantly different from the Unfermented Sample (Day 1) at p < 0.05.

Values with superscript (^ǂ^) are significantly different from the Ascorbic acid (Standard compound) at p < 0.05.

## 4 Discussion

### 4.1 Effect of fermentation on the phytochemical of unripe plantain fruit

This study explored a chance inoculation of fermenters to ferment unripe plantain pulp for 13 days. Fermentation is solely driven by microbial activities that usually result in macromolecule degradation, transformation and modification [2,[13], [14], [15]]. The bacterial proliferation rate progressed slowly for the first 48 h (days 1–3); afterwards, the proliferation rate became more rapid until day 7, Fig. 1a. The fermenters' proliferation rate peaked from days 7–10 (stationary proliferation phase), and a decline in the proliferation rate was recorded from day 11 to the end of this study. The sample's temperature ranged between 29.50 °C on day 1 and 27.00 °C on day 13, while the pH ranged between 6.82 and 5.71, Fig. 1b. The percentage changes in the temperature showed a significant rise and drop within the first four days. They maintained a steady decrease until day 9, when they attained a stable temperature range till day 13. Contrary to the change in temperature, pH showed no noticeable change until day 11 and sustained a steadily increased acidity till the end of the fermentation, Fig. 1b.

Protein fragmentation and production during fermentation have been reported to process bioactive effects and are considered bioactive peptides (BAPs) [43,44]. The protein content of the fermented hydroalcoholic extract of unripe plantain in Fig. 1c showed that fermentation significantly increased the protein content of the extract steadily from day 1 to day six and showed a non-significant staggering pattern until day 13. Nikhata et al. [44] reported that fermentation increases fermented food's nutritional and digestible protein content. Also, the protein content from days 5–13 was significantly (p < 0.05) higher than that of the unfermented sample (day 1). The increased protein formation by bacteria might be favoured by the decrease in pH and reaction temperature as the Pearson correlation of these factors was significantly high (Fig. 1h); the point at which the pH and temperature changes had a p-value 1.00. The increased protein content recorded in this work may not be unrelated to the formation of some amide-containing compounds, which have been reported to possess antioxidant and antimicrobial properties [45].

The ascorbic acid content of the fermented samples in Fig. 1d showed a significant increased (p < 0.05) till day 3, and no significant change was noted until day 9, which was not significantly (p > 0.05) different from the unfermented sample (day 1). In Fig. 1e, fermentation significantly reduced (p < 0.05) flavonoid content in plantain pulp extract within 24 h; it is noteworthy to state that the flavonoid level remained insignificantly (p > 0.05) altered from day 2 to day 13. Li et al. [46], reported a closely similar pattern in the flavonoid content of green banana pulp fermented wine. Contrary to the flavonoid, the phenolic content was significantly (p < 0.05) increased by fermentation daily until day 5. The phenolic content from day 7 to day 13 was not altered significantly (Fig. 1f); however, it was significantly higher than the unfermented sample. According to Li et al. [46], an increased phenolic content was recorded in green banana peel wine, while the pulp of the same fruit wine showed a reduction during fermentation. The fermenter's activities have been ascribed to be responsible for the increased phenolic contents either by converting other macromolecules or/and dissociating phenols from their conjugates [47].

### 4.2 Pearson's correlation of physical and phytochemical changes of 3 days interval fermentation

The Pearson correlation *heatmap* in Fig. 1 (g – l) showed the associations between the change in physical and phytochemical content of the hydroalcoholic extract of plantain fruit per fermented periods of Days 1–3, 3–5, 5–7, 7–9, 9–11 and 11–13. In this study, the Pearson correlation (p-value ≤ −0.95 or ≥ 0.95) was only considered negatively or positively correlated, corresponding to a 95 % confidence level. In Fig. 1g, the microbial activities showed no association with the change in temperature and pH; however, temperature and pH change were inversely correlated. The proteins, ascorbic acid and phenols were positively correlated with the fermenters' proliferation rate within the first three days (days 1–3). The inverse relationship between the pH and temperature has been ascribed to the fermenter's activities and growth rate during fermentation [48]. The physical changes within the same period had no significant correlation with the four phytochemicals studied in this work.

Between days 3 and 5 (Fig. 1h), The fermenters, proliferation rate was positively correlated with the protein only. There was a negative correlation between the temperature change and flavonoid level, and the flavonoid had a positive association with the pH change. Also, there was a positive correlation between the temperature change and the protein content, while the change in the total protein content showed a negative correlation with the pH change. Nkhata et al. [49] reported that fermenters' actions due to the utilisation and loss of carbohydrates may account for the increase in protein content of fermented samples within the first 48 h of the fermentation process.

The pH changes between days 5 and 7 (Fig. 1i) were negatively correlated with fermenters’ proliferation rate. Also, the temperature change showed a negative correlation with the flavonoid content. In Fig. 1j (days 7–9), the bacteria proliferation was inversely correlated to the total protein content of the methanolic extract of the fermented unripe plantain pulp. No correlation was observed between the physical and phytochemical constituents of the fermenting sample on days 7–9. In Fig. 1k, the microbial cell count was positively correlated to the phenolic content; an inverse association was observed between the temperature change and the change in flavonoid content. On days 11–13 (Fig. 1l), the microbe proliferation rate showed weak positive and negative correlations with the changes in temperature and pH for the fermented sample, respectively. However, the change in flavonoid content showed a positive correlation with the microbial load of the fermented sample. It is well established that microbial activities are consequential to temperature and pH; however, these weak associations suggest bacteria actions may not be dependent on their proliferation rate in an isothermal system. that the period with a decrease in temperature (reduced fermenters activities depreciate), the total phenols content remains on the rise suggesting that a prolonged fermentation period may favour an increase in phenolics compared to other phytochemicals estimated in this study. Knez et al. [50] stated that ascorbic acid content in fermented samples is inversely proportional to the fermentation time in some selected vegetables and legumes.

### 4.3 ^1^H NMR-based metabolomics and compound identification

NMR spectroscopy provides reliable and reproducible information regarding the chemical environments of compounds present in crude extracts (Mlynárik, 2017). The PCA sufficiently separated the 13-day fermentation samples of *M. paradisiaca* fruits. The separation was further explored by OPLS-DA, which revealed the discriminant chemical shift of compounds that significantly contributed to the separation of group 1 from 2. It could be inferred from the clusters that day 1 to day 6 and day 13 have standard features, while the features of day 7 to day 12 distinguished them as extended fermentation samples. Notably, most of the chemical shift that separates group 1 samples from group 2 is in the upfield region. Thus, it may be due to insignificant differences in the aromatic and highly overlapped sugar regions not sufficient to distinguish between the two groups. Based on the findings of the biological studies, groups 1 and 2 of the OPLS-DA score plot may be assigned active and inactive groups, respectively.

Although there were limited peaks in the aromatic region of both spectra, day 9 had more peaks than day 2, suggesting that extended fermentation might have increased the production of phenolic metabolites. Phenolic compounds, gallic acid and methyl gallate were annotated upon a comparison of their NMR data with literature values. The observed reduction in peaks and peak intensity sugar region of day 9 compared to day 2 confirms the expected biotransformation of sugars during fermentation. A monosaccharide sugar, mannose was further identified alongside some primary amino acids and lipids of therapeutic importance, among which is N-acetyl dihydrosphingosine, an analogue of the ceramides that are known for their tumour necrosis factor (TNF-*α*) signalling [51].

The UPLC-QTOF-MS is esteemed for its unparalleled precision and depth of information in unravelling the complexities of natural crude extracts [52]. Structure determination from the spectral data afforded the compounds N-acetylhistidine, leucyl-glycyl-glycine, akodionine, 4-aminophenylalanine, pyrrolam B, streptanoate, phytosphingosine, palmitoylputrescine and ricinoleic acid which were exclusive to day 9. One of the aims of this current study is the biomodification of phytochemicals for enhanced antioxidant, anti-inflammatory and antidiabetic potentials. The formation of the compounds mentioned above demonstrated that most of these compounds are metabolites of some fermenters' metabolic pathways. Their relevance as antioxidants, anti-inflammatory and some enzyme inhibitors have been reported, including N-acetylhistidine and 4-aminophenylalanine have been associated with propionic acid bacteria [53]. A previous study further reported the presence of *Propionibacterium* in fermented plantain pulp samples [13]. Prelegonmycin A and pyrrolam B are alkaloids linked to one of the fermentation products [54]. Similarly, streptanoate has been found in *Streptomyces sp.* which has been reported as a potent anticancer agent [55].

### 4.4 Bacterial isolation, identification and carbon utilisation profiling

Fermentation is a bacterial action-driving process coordinated by factors like pH, temperature, type and abundance of substrates. In this study, the morphology and biochemical characteristics (Table 3a) of the bacteria isolate showed the presence of rod-like, gram-negative and gram-positive *Escherichia coli* and *Propionibacterium* spp, respectively. Also, these organisms showed different forms of respiration, while *E. coli* respire aerobically, the *Propionibacterium* spp. was anaerobic. Pathogenic tests on the two bacteria suggest that *E. coli* may be non-pathogenic, while *Propionibacterium* spp appeared opportunistic. This observation agrees with the studies of Nkhata et al. [44], who reported that some enteric *E. coli* are non-pathogenic. The enzyme assay profiling of the two bacteria showed that they possess acid phosphatase, catalase, tryptophan deaminase and nitrate-reducing enzymes, while arginine dehydrolase, lecithinase, lipase, ornithine decarboxylase and urase were absent in both organism, Table 3b. The carbon source utilisation profiling in Table 3c showed that *E. coli* utilises more sugar as a carbon source than *Propionibacterium* spp. However, both bacteria could not ferment cellobiose, glycogen, Inositol and raffinose. Sekar et al. [56] reported that *E. coli* lacks the potential to ferment cellulose, and Delgado et al. [23] showed that some species of *Propionibacterium* cannot utilise selected carbon sources like glycogen and raffinose.

### 4.5 Antioxidant profiling of fermentation products of unripe plantain fruits

Antioxidant properties of the fermentation on unripe plantain pulp are shown in Table 4, and the significance level was considered at p < 0.05. The DPPH inhibition potential of the fermented samples was significantly higher than the unfermented (Day 1) except on days 6, 10 and 11, which were not significantly different from the unfermented extract. Cuellar Alvarez et al. [57] reported that after day 6 of fermenting, a comparison between the fermented samples and the ascorbic acid (standard antioxidant) showed that all the fermented extracts inhibited DPPH more significantly than the ascorbic acid. The ferric-reducing potential (FRAP) recorded in the fermented unripe plantain pulp extracts was not significantly different from the unfermented extract except on days 8 and 12, which were significantly lower. Ferric-reducing antioxidant power of the fermented and unfermented plantain extracts was not significant from the ascorbic acid except at day 8, which was significantly lower. According to the study of Cuellar Alvarez et al. [57], the DPPH and FRAP inhibition capacities of fermented Cupuassu beans declined due to the reduction in some of its phytochemicals and, in our study, a significant increase in both the phenolics and flavonoids contents was within this period.

The effect of fermentation time (days 2–13) on the hydrogen peroxide scavenging potential of unripe plantain pulp extracts was not significantly altered (p < 0.05) when compared to the unfermented extract (Table 3). A comparison between the ascorbic acid and the fermented samples revealed that days 10, 11 and 12 were not significantly different from the ascorbic acid. In contrast, the unfermented and the other fermented samples (days 2–9 and 13) showed a better hydrogen peroxide scavenging capacity. Lipid peroxidation has been reported as the end-product of the consequence of free radicals-overwhelmed biological systems. *In-vitro* lipid peroxidation assay in this work showed that unfermented, fermented and ascorbic acid were not significantly different (p > 0.05) except for days 8 and 9, which had a significantly higher lipid peroxidation inhibition capacity than both unfermented (day1) and the ascorbic acid.

A relative evaluation of the overall antioxidant capacity between the unfermented and fermented unripe plantain fruit-pulp extract (Table 3) revealed that day 9 had the highest antioxidant potential. Hence, it could be ascribed to some unique compounds identified by the ^1^H NMR and UPLC-QTOF-MS data in this study (Tables 1a and 1b). Also, phenols characterisation of plantain-based dough meal by Oluwajuyitan et al. [11] showed that gallic acid and a few other phenolics are present in unripe plantain pulp; however, the fermentation process at day nine might have increased these polyphenols content by either conversion of some sugar moiety or/and hydrolysed some of the bound phenols to their free-state [58]. Hence, it may account for the significant increase in the antioxidant capacity of the day 9 sample. Furthermore, the tripeptide (leu-gly-gly) present in the day 9 sample is likely to play a critical role in the enhanced antioxidant activity due to the structural activity, which has been reported to form a stable complex with pro-oxidant Cu (II) [59] and the aromatic lipid (ricinoleic acid), which review has shown to possess an antioxidant property [60]. The combined antioxidant effect of N-acetylhistidine and a few phenolics phytoconstituents is well documented in the inhibition of lipids peroxidation in plants and oils [61,62].

### 4.6 Effect of fermentation on the in-vitro anti-inflammatory and α-amylase inhibition profiles of unripe plantain fruits

The effect of fermentation on the anti-inflammatory and α-amylase inhibition evaluated in this work was reported in Table 3. The protein denaturation inhibition assay revealed that days 2 and 3 had significantly higher protein denaturation inhibition potentials than the unfermented (day 1). However, the unfermented (day 1) protein denaturation inhibition potential and days 3–13 were not significantly different from the ascorbic acid. Nitric oxide synthesis via endogenous and exogenous means is one of the key inflammatory markers in the biological system [63]. The nitric oxide scavenging potential of the unfermented, fermented samples and ascorbic acid was not significant (p < 0.05), except on days 9 and 11, which showed a more robust nitric oxide scavenging capacity over both unfermented and the ascorbic acid. The dual role of nitric oxide as a component of oxidative stress (reactive nitrogen species) and inflammatory signalling molecule is well established; hence, nitric oxide scavenging via direct (antioxidants) or/and indirect (inhibition of endogenous nitric oxide synthesis) has been considered an efficient anti-inflammatory mechanism [64]. The significant scavenging of nitric oxide recorded on day nine might be via the additive antioxidant channel via some of the nine compounds unique to day 9 (listed in Table 1b). The synergistic antioxidant effect of the derived lipids - phytosphingosine was reported in a study by Zhao et al. [65], while Johnson & Coutinho [66] showed that phytosphingosine and other constituents of *Sphaerostephanos arbuscula* chloroform extract might be responsible for its anti-inflammatory potential. Also, ricinoleic acid [67] might significantly contribute to the scavenging effect as ricinoleic acid has been reported to inhibit inflammation in oedema subjects independently [68].

Alpha-amylase antagonists are the frontline tools for managing pre-diabetes and T2DM conditions [69]. The amylase activity inhibition assayed in this study revealed that the fermented sample on days 9, 10 and 11 showed a significantly higher (p < 0.05) α-amylase activity antagonistic power over the acarbose (*standard amylase inhibitor*) and the unfermented sample (day 1). While a study by Grote et al. [70] reported no amylase inhibition potential in the pyrrolam A and B compounds identified in this work yet, a substantial amylase inhibition potential was recorded on days 9–11, and this could be ascribed to the synergistic role of 3-Methoxy-4-hydroxyphenyl 6-*O*-(3,4,5-trihydroxybenzoyl)-beta-D-glucopyranoside and gabalid, reported by Remok et al. [71] and other metabolites especially phytosphingosine [72] which is unique to the day 9 sample. Martins et al. [73] proposed that the antioxidant potential of phenolics enables them to act as amylase inhibitors. Ricinoleic acid, the major fatty acid in Castro oil, is well-reported as an antidiabetes agent in both *in-vitro* and *in-vivo* studies [74]. Significant biotransformation and improved antioxidant, anti-inflammatory and antihyperglycemic profiles seen in unripe *M. paradisiaca* fruit pulp in this study correlated with the prolonged fermentation time. It is corroborated by Feng et al. [75] and Li et al. [46], who reported that seven days or more of fermentation time promotes the chemical profile and other bioactivities of Qingzhuan tea and green banana pulp wine, respectively.

### 4.7 Study limitations

The current study adopted morphological identification of the fermenters rather than genotypic identification. Although, Propionibacterium morphology and genotyping were reported recently [[21], [22], [23]}.

## 5 Conclusion

This study evaluated the correlation between the fermentation time, physicochemical conditions, and unripe M. paradisiaca pulp metabolite patterns via *in-vitro* assay methods. The results revealed that *E. coli* and *Propionibacterium* are the bacteria morphologically and biochemically identified to be responsible for the fermentation of the unripe plantain pulp sample, and proliferation and activities were at their peak from days 7–10. The phenolic contents are positively correlated with fermentation time, while flavonoids and ascorbic acids were inversely associated with the fermentation time. The ^1^H NMR fingerprint and chemometrics revealed a closely related chemical shift in fermentation time (days 2–6) with relatively even bioactivity with the unfermented. In contrast, the fermentation periods (days 7–12) possess enhanced bioactivity and are closely related. Also, the formation of some phenolics, a tripeptide, lipid and amino acid derivatives (3-methoxy-4-hydroxyphenyl 6-*O*-(3,4,5-trihydroxybenzoyl)-beta-D-glucopyranoside, N-acetylhistidine, urocanic acid, 2-aminocyclopentanecarboxylic, leucyl-glycyl-glycine, akodionine, rubrenoic acid, prelegonmycin A, 4-Aminophenylalanine, pyrrolam B, streptanoate, cystodienoic acid, phytosphingosine, palmitoylputrescine, ricinoleic acid gabalid) were annotated in the ^1^H NMR spectra and UPLC-QTOF-MS data. These compounds were noted to be responsible for the improved bioactivities recorded in fermented methanolic extracts of the unripe plantain pulp. Thus, this study has established the potential of a 9-day fermented unripe plantain fruit methanolic extract as a potent nutraceutical agent against diabetes conditions and their associated complications, including cancer. It has opened a gate for *in-vivo* study of other metabolic disorders, including cardiovascular and neurodegenerative diseases, as diabetes mellitus propagates the risk of these degenerative disorders.

## CRediT authorship contribution statement

**Atunnise Adeleke Kazeem:** Writing – original draft, Methodology, Investigation, Formal analysis, Conceptualization. **Olusola Bodede:** Writing – original draft, Methodology, Formal analysis. **Adewuyi Adewale:** Writing – review & editing, Supervision. **Vinesh Maharaj:** Writing – review & editing, Validation, Data curation. **Gerhard Prinsloo:** Writing – review & editing, Validation, Resources. **Bamidele Adewale Salau:** Writing – review & editing, Supervision, Conceptualization.

## Declaration of competing interest

The authors declare that they have no known competing financial interests or personal relationships that could have appeared to influence the work reported in this paper.
