# Supplementary material for: Metabolomics and in-vitro bioactivities studies of fermented Musa paradisiaca pulp: A potential alpha-amylase inhibitor
Source: Heliyon. 2024 Jan 19;10(3):e24659. doi: 10.1016/j.heliyon.2024.e24659 (PMC10839803; doi:10.1016/j.heliyon.2024.e24659)
Supplement: Multimedia component 1 [file mmc1.docx]

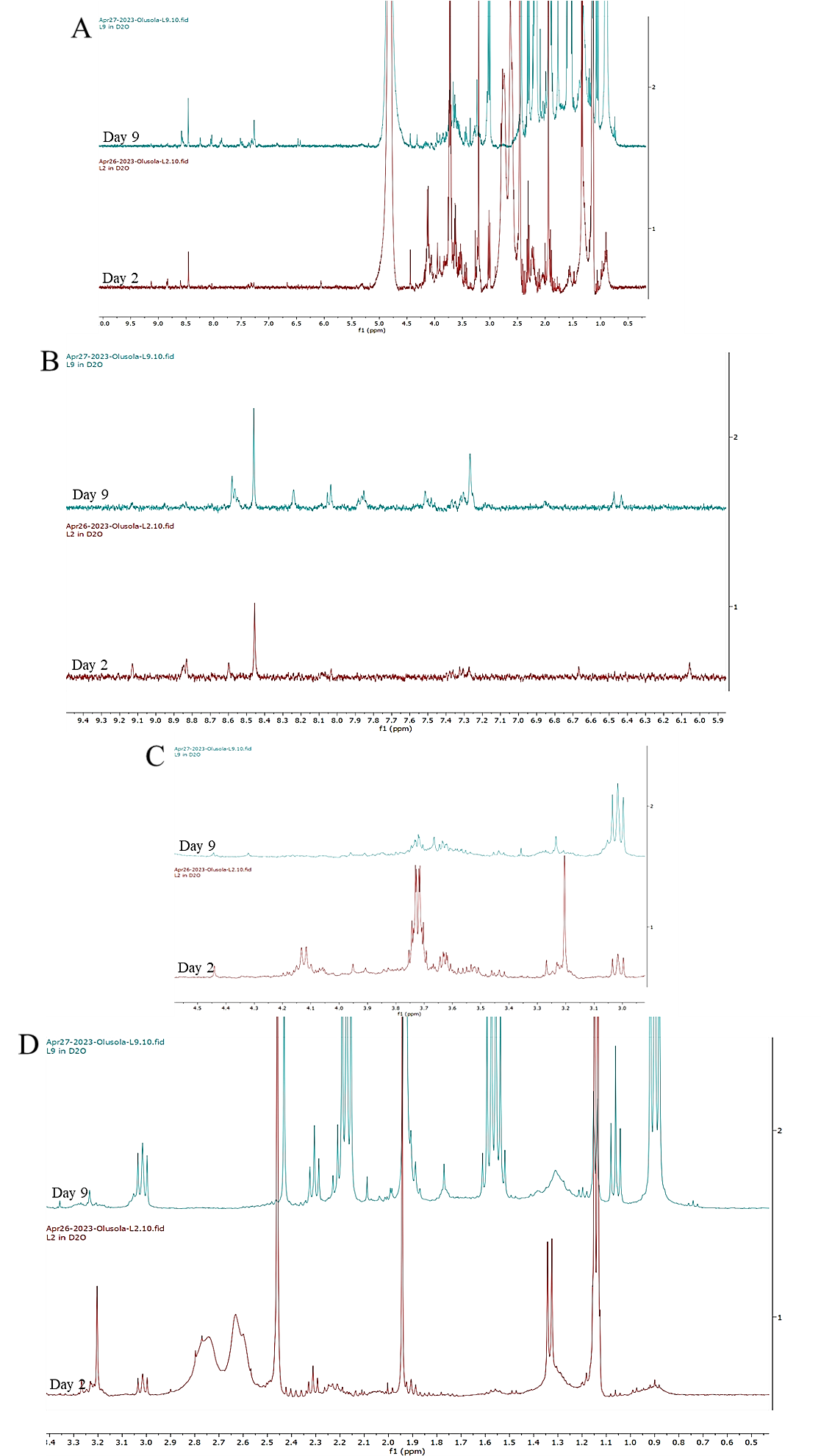


**Figure 3.** ^1^H NMR spectra of day 9 compared to day 2. A: Full spectra, B: Expanded (6.0 – 9.5 ppm), C: Expanded (3.0 – 4.5 ppm), D: Expanded (0.5 – 3.3 ppm)
